# Development of genus-specific universal primers for the detection of flaviviruses

**DOI:** 10.1186/s12985-021-01646-5

**Published:** 2021-09-15

**Authors:** Tomo Daidoji, Ronald Enrique Morales Vargas, Katsuro Hagiwara, Yasuha Arai, Yohei Watanabe, Keisuke Nishioka, Fumi Murakoshi, Kotaro Garan, Hiroki Sadakane, Takaaki Nakaya

**Affiliations:** 1 Department of Infectious Diseases, Graduate School of Medical Science, Kyoto Prefectural University of Medicine, Kyoto, 602-8566 Japan; 2 Department of Medical Entomology, Faculty of Tropical Medicine, Mahidol University, Bangkok, Thailand; 3 Veterinary Virology, School of Veterinary Medicine , Rakuno Gakuen University, Hokkaido, 069-8501 Japan

**Keywords:** Universal primer, Flavivirus, RT-PCR, Arthropod

## Abstract

**Background:**

Flaviviruses are representative arboviruses carried by arthropods and/or vertebrates; these viruses can pose a public health concern in many countries. By contrast, it is known that a novel virus group called insect-specific flaviviruses (ISFs) also infects arthropods, although no such virus has yet been isolated from vertebrates. The characteristics of ISFs, which affect replication of human-pathogenic flaviviruses within co-infected mosquito cells or mosquitoes without affecting the mosquitoes themselves, mean that we should pay attention to both ISFs and human-pathogenic flaviviruses, despite the fact that ISFs appear not to be directly hazardous to human health. To assess the risk of diseases caused by flaviviruses, and to better understand their ecology, it is necessary to know the extent to which flaviviruses are harbored by arthropods.

**Methods:**

We developed a novel universal primer for use in a PCR-based system to detect a broad range of flaviviruses. We then evaluated its performance. The utility of the novel primer pair was evaluated in a PCR assay using artificially synthesized oligonucleotides derived from a template viral genome sequence. The utility of the primer pair was also examined by reverse transcription PCR (RT-PCR) using cDNA templates prepared from virus-infected cells or crude supernatants prepared from virus-containing mosquito homogenates.

**Results:**

The novel primer pair amplified the flavivirus NS5 sequence (artificially synthesized) in all samples tested (six species of flavivirus that can cause infectious diseases in humans, and flaviviruses harbored by insects). In addition, the novel primer pair detected viral genomes in cDNA templates prepared from mosquito cells infected with live flavivirus under different infectious conditions. Finally, the viral genome was detected with high sensitivity in crude supernatants prepared from pooled mosquito homogenates.

**Conclusion:**

This PCR system based on a novel primer pair makes it possible to detect arthropod-borne flaviviruses worldwide (the primer pair even detected viruses belonging to different genetic subgroups). As such, an assay based on this primer pair may help to improve public health and safety, as well as increase our understanding of flavivirus ecology.

**Supplementary Information:**

The online version contains supplementary material available at 10.1186/s12985-021-01646-5.

## Background

Flaviviruses, which are representative arboviruses that infect arthropods and/or vertebrates, can cause public health problems worldwide [1]. Generally, the genus flavivirus is divided into four groups: (i) mosquito-borne flaviviruses, (ii) tick-borne flaviviruses, (iii) insect-specific flaviviruses (ISFs), and (iv) no known vector (NKV) flaviviruses [2–5]. Of these, mosquito-borne and tick-borne flaviviruses, which include Dengue virus (DENV), West Nile virus (WNV), Yellow fever virus (YFV), Japanese encephalitis virus (JEV), Zika virus (ZIKV), and Tick-borne encephalitis virus (TBEV), are pathogens that play an important role in human diseases such as encephalitis, fever, and hemorrhagic fever [5, 6]. A novel virus group, ISFs was defined recently; this group also infects arthropods, although no ISF has yet been isolated from any vertebrate. ISFs comprise two subgroups: classical ISFs [7–23] and dual-host affiliated ISFs [24–32]. Because some ISFs enhance or suppress replication of human-pathogenic flaviviruses within co-infected mosquito cells or mosquito laboratory colonies [19, 33–36], attention should be paid to both ISFs and human-pathogenic flaviviruses, even though ISFs appear not to be directly hazardous to human health. Therefore, to better assess the risk posed by flavivirus-induced diseases in humans, and to increase our understanding of the ecology of flaviviruses, we need to examine the distribution and diversity of flaviviruses using a broad detection system that can detect a wide range of these viruses in arthropods, which are known reservoirs.

Reverse transcription PCR (RT-PCR) is a well-established method used to detect/identify the genomes of micro-organisms such as bacteria, fungi, and viruses [37–42]. The RT-PCR method for detecting flaviviruses is well-established and used routinely by many laboratories [43–59]. Viruses belonging to the flavivirus genus have a positive-sense, single-stranded RNA genome (9.2–11 kbp), which encodes three structural (C, prM, and E) and seven non-structural (NS) proteins (NS1, NS2A, NS2B, NS3, NS4A, NS4B, and NS5) [5]. To date, different research groups have proposed sets of universal primer pairs designed to enable use of RT-PCR to detect NS proteins encoded by a range of flavivirus genomes [54–59]. However, although these primer pairs can efficiently detect virus species that are closely related, they are not very good at detecting more distantly related species or completely different species. In particular, these primer pairs may not detect more recently identified flavivirus strains. For example, classical ISFs which comprise a novel virus group, show high levels of genetic deviation from the three major subgroups, i.e., mosquito-borne flaviviruses, tick-borne flaviviruses, and NKV flaviviruses [2–5]. Thus, it is likely that the above-mentioned universal primers may not detect classical ISFs.

Therefore, the purpose of this study was to develop a detection system that recognizes flavivirus NS5 sequences broadly across the flavivirus genus. First, we identified conserved sites within the NS5 region of the flavivirus genome; such sites are conserved among many different subgroups of viruses, including mosquito -borne flaviviruses, tick-borne flaviviruses, ISFs, and NKV flaviviruses. We then designed a universal primer pair that amplifies this genome segment. To confirm the practical utility of this primer pair, we amplified the NS5 region from eight different flavivirus genomes (mosquito -borne flaviviruses, tick-borne flaviviruses, and ISFs). In addition, we used the primer pair to detect the flavivirus genome specifically in live virus-infected cells (a mosquito-derived cell line). Finally, we used the primer pair to amplify the flavivirus NS5 coding region from crude mosquito supernatants prepared from pooled mosquito homogenates.

## Materials and methods

### Primer design

**Fig. 1:**
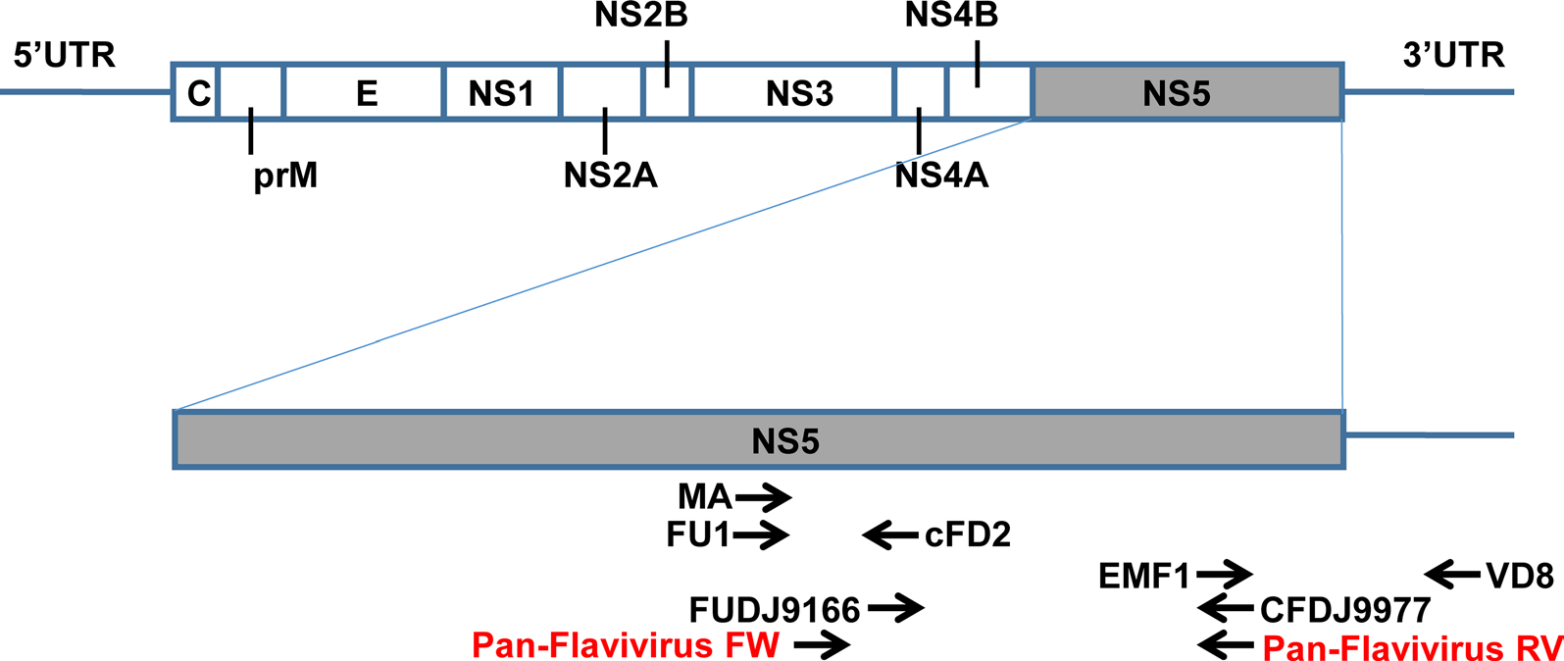
Binding sites of the respective universal primer pairs in the flavivirus genomes. The name of each primer pair is the same as that reported in the original articles: FU1/cFD2 [56, 57], MA/cFD2 [57], EMF1/VD8 [55], FUDJ9166/CFDJ9977 [54], and Pan-flavivirus FW/RV (this article)

**Fig. 2:**
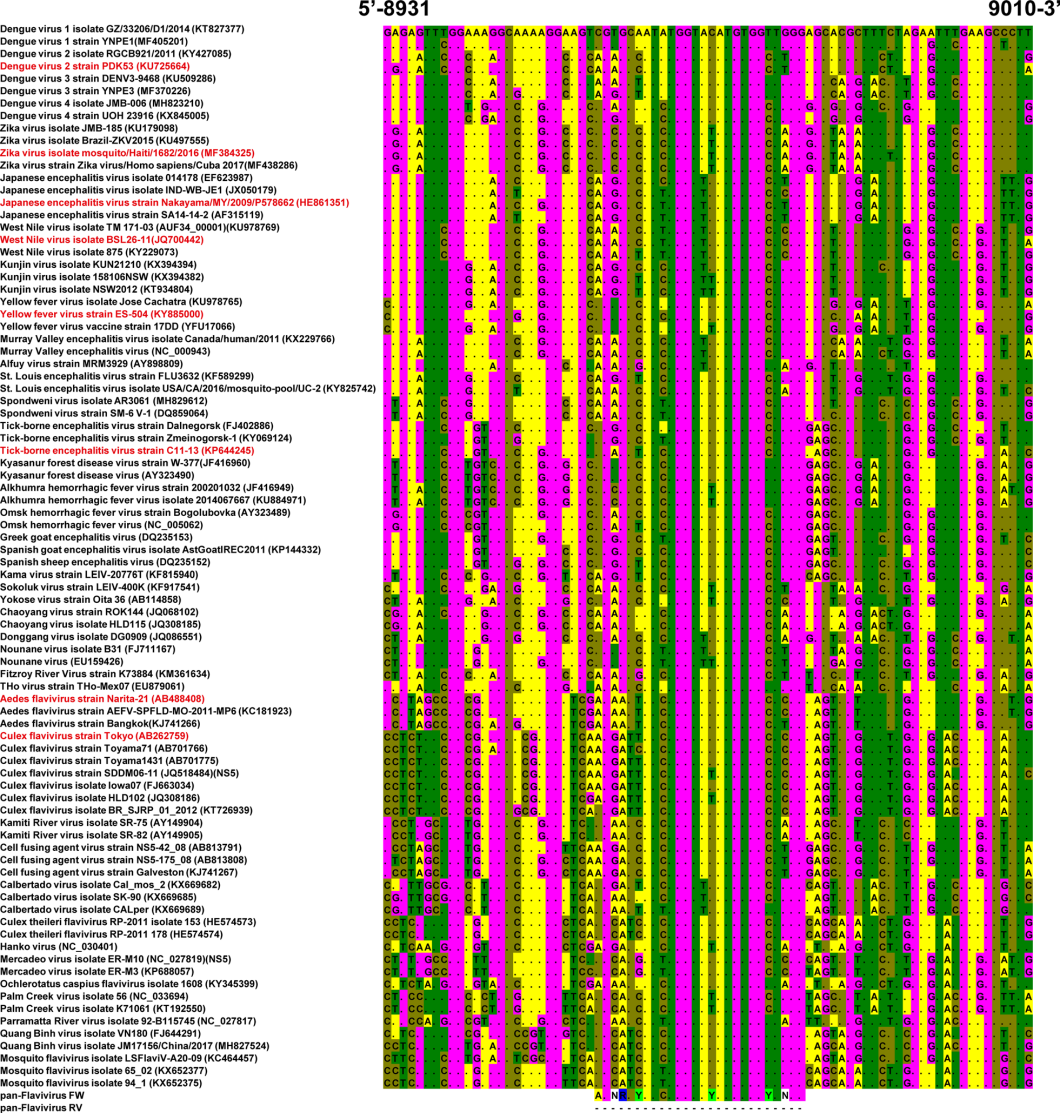

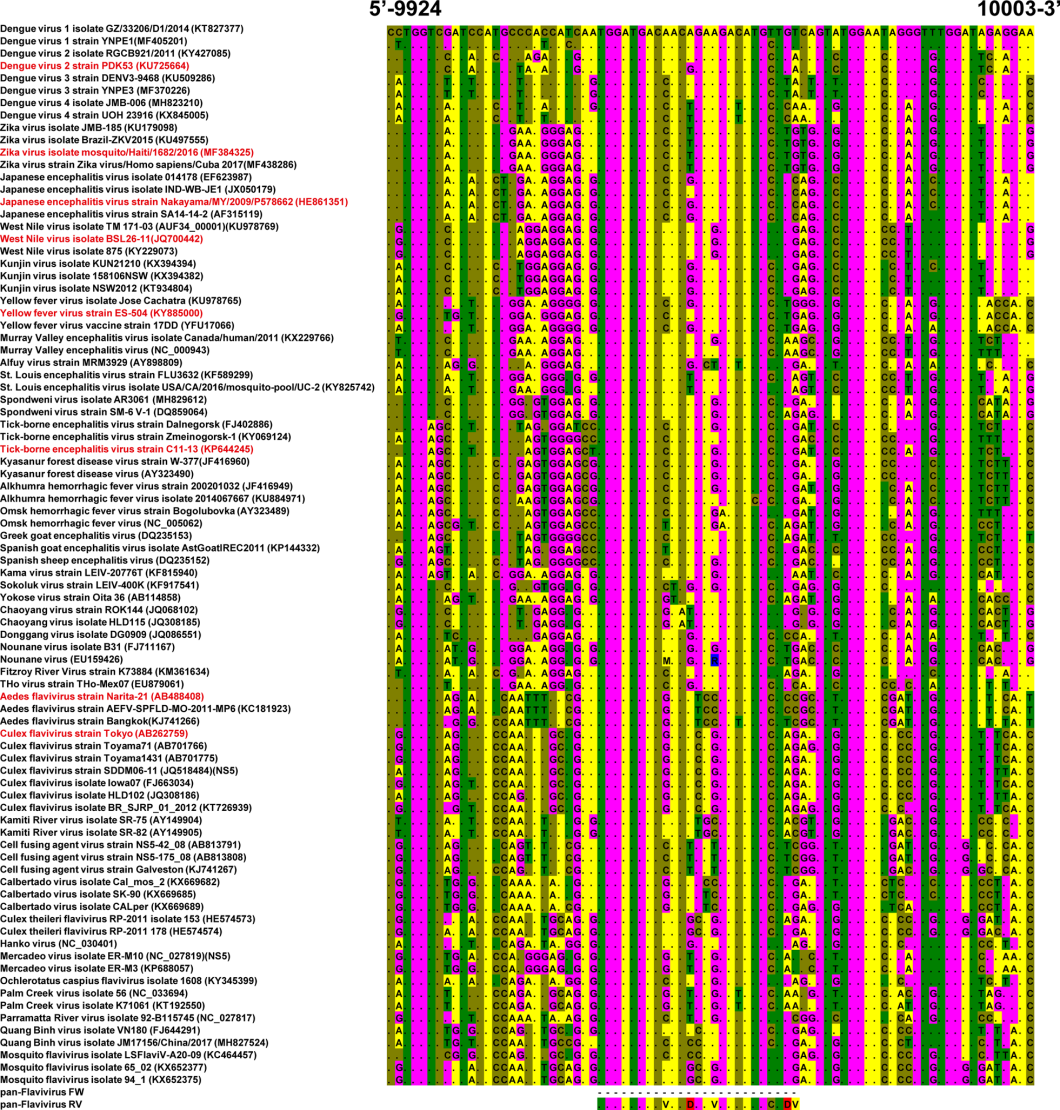
Multiple alignment of NS5 sequences. NS5 sequences from mosquito -borne flaviviruses, tick -borne flaviviruses, NKV flaviviruses, and some dual-host affiliated ISFs, classical ISFs, and other unclassified flaviviruses, were aligned using Clustal W. The site on which the primer design was based is also shown. The numbering of the nucleotides on the 5′ and 3′ sides corresponds to that of DENV 1 GZ/33206/D1/2014 (accession KT827377). Dots indicate identicality with the consensus sequence used for alignment. The colors indicate an individual base: “A”, yellow; “T”, green; “G”, magenta, and “C”, olive for the nucleotides in the NS5 sequences of flaviviruses; “V,” yellow; “D”, red; “R”, blue; “N”, white; “Y”, bright green for the nucleotides in the primer sequences; and “R”, blue for the nucleotides in the NS5 sequences of the Nounane virus EU159426. The names in red are reference strains, the sequences of which were used as PCR templates in this study

First, a universal primer pair (denoted as pan-flavivirus FW/RV; see the Table 1) was designed to target the NS5 coding region, which is conserved across different species of flavivirus. This was done by performing multiple alignments of NS5 region nucleotide sequences from mosquito-borne flaviviruses, tick-borne flaviviruses, ISFs, and NKV flaviviruses (Fig. 1 and Fig. 2). Sequence alignment was conducted using CLUSTALW and the results were visualized using MEGA [60] version 10.1.7. Although the predicted amplicon size generated by RT-PCR using this universal primer is similar among species, it remains species-dependent; the predicted amplicon sizes generated from the viral genome template used in this study are as follows: 1018 bp for DENV 2 PDK53 (GenBank accession KU725664); 1021 bp for ZIKV mosquito Haiti/1682/2016 (GenBank accession MF384325); 1027 bp for JEV Nakayama MY 2009 P578662 (GenBank accession HE861351); 1027 bp for WNV isolate BSL26-11 (GenBank accession JQ700442); 1027 bp for YFV strain ES-504 (GenBank accession KY885000); 1018 bp for TBEV strain C11-13 (GenBank accession KP644245); 1009 bp for Aedes flavivirus Narita-21 (GenBank accession AB488408); and 1009 bp for Culex flavivirus Tokyo (GenBank accession AB262759).

**Table 1:** Sequences of the oligonucleotide primer pairs tested in this study

Primer name	Sequence (5′ to 3′)	Product size^a^	References
FU1	TACAACATGATGGGAAAGAGAGAGAA	266 bp	Kuno et al., 1998[56–58]
cFD2	GTGTCCCAGCCGGCGGTGTCATCAGC		
MA	CATGATGGGRAARAGRGARRAG	261 bp	Kuno et al., 1998[57, 58]
cFD2	GTGTCCCAGCCGGCGGTGTCATCAGC		
EMF1	TGGATGACSACKGARGAYATG	627 bp	Pierre et al., 1994 [55, 58],
VD8	GGGTCTCCTCTAACCTCTAG		
FUDJ9166	GATGACACAGCAGGATGGGAC	832 bp	Chang et al., 1994[54, 58]
CFDJ9977	GCATGTCTTCCGTCGTCATCC		
Pan-flavivirus FW	AGNRCYATCTGGTAYATGTGGYTNGG	1018 bp	This article
Pan-flavivirus RV	BHAGCATGTCBTCHGTBGTCATCCA		

^a^Size is based on the PCR amplicon generated from a genome template of DENV 2 PDK53 (accession KU725664)

### PCR amplification of flavivirus NS5 regions

To confirm the utility of the novel universal primer pair (denoted as pan-flavivirus FW/RV), it was used to amplify the NS5 region of representative virus strains belonging to the genus flavivirus (i.e., DENV, ZIKV, JEV, WNV, YFV, TBEV, Aedes flavivirus, and Culex flavivirus) in a PCR; other published universal primer pairs were tested for comparison [54–58]. Individual strains isolated in the 2000s (except JEV) were used to confirm that the universal primers can detect more recent viruses. The following oligonucleotides encoding the NS5 coding region (not the whole sequence) and the 3’-UTR were synthesized artificially (Eurofins Genomics, Tokyo, Japan) for use as templates in the PCR assay: DENV 2 PDK53 (corresponding to bp 8923–10602; accession KU725664); ZIKV mosquito Haiti/1682/2016 (corresponding to bp 8151–10808; accession MF384325); JEV Nakayama/MY/2009/P578662 (corresponding to bp 9036–10770; accession HE861351); WNV BSL26-11 (corresponding to bp 8164–11029; accession JQ700442); YFV ES-504 (corresponding to bp 8120–10699; accession KY885000); TBEV C11-13 (corresponding to bp 8149–10838; accession KP644245); Aedes flavivirus Narita-21 (corresponding to bp 7903–10760; accession AB488408); and Culex flavivirus Tokyo (corresponding to bp 7961–10837; accession AB262759). The PCR assay was performed using DNA polymerase (KOD plus Neo; Toyobo, Osaka, Japan) plus the above primers. Each 25 µl reaction mixture comprised 10 × PCR buffer (2.5 µl), 2 mM dNTPs (2.5 µl), 25 mM MgSO_4_ (1.5 µl), 10 µM forward primer (0.75 µl), 10 µM reverse primer (0.75 µl), template DNA (1 µl) or distilled water (1 µl; negative control), KOD plus Neo polymerase (0.5 µl), and distilled water (15.5 µl). PCR was conducted using a TaKaRa PCR Thermal Cycler Dice (Takara Bio, Shiga, Japan). The cycling conditions were as follows: initial denaturation at 94 °C for 2 min, followed by 35 amplification cycles (denaturation at 98 °C for 10 s, annealing at 60 °C for 30 s, and extension at 68 °C for 15 or 30 s), and a final extension at 68 °C for 2 min. The extension time was dependent on the primer pair used and on the size of the amplified product, i.e., 15 s for FU1/cFD2 and MA/cFD2, and 30 s for all other primer pairs (please see the Table 1). The amplicons produced by the PCR assay were confirmed by running the products on 1.5% agarose gels, followed by staining with ethidium bromide. The PCR product amplified by the novel universal primers (pan-flavivirus FW/RV) was sequenced.

### Amplification of NS5 coding region from infected cells using the RT-PCR method plus the newly designed universal primer pair

Next, the new primer pair was used to detect the flavivirus genome in cells infected with JEV. Here, the NS5 coding region was amplified from cell lysate samples to confirm the specificity of the primer pair for the flavivirus genome. The mosquito cell line (C6/36), which is derived from *Aedes albopictus*, was cultured (4.0 × 10^5^ cells/well) in 12-well plates at 28 °C in Eagle’s minimum essential medium (Wako Pure Chemical Industries, Osaka, Japan) containing 5% fetal bovine serum and standard antibiotics (penicillin (100 units/mL), streptomycin (100 µg/mL), and amphotericin B (250 ng/mL)). Cells were washed twice with PBS (supplemented with calcium/magnesium; PBS( +)) and then infected with a JEV live attenuated pig vaccine (m strain) (Kyoto Biken Laboratories, Uji, Kyoto) at a multiplicity of infection (m.o.i.) of 0.2, 0.02, and 0.002. Controls were mock-infected with PBS ( +). The m.o.i. was based on the cell number and the titer of the virus used (the virus titer was determined based on the TCID_50_, which was obtained using C6/36 cells and the Reed-Muench method [61]). Virus was resuspended in PBS ( +) and incubated with the cells for 2 h at 28 °C. After removing the virus solution, the cells were washed twice with PBS ( +) and cultured at 28 °C in Eagle’s minimum essential medium (Wako Pure Chemical Industries) containing 2% fetal bovine serum and standard antibiotics. Total RNA was extracted from infected cells using a NucleoSpin® RNA Plus (Takara Bio, Shiga, Japan) kit, and single-stranded cDNA was synthesized from the extracted RNA (0.2 µg) using random primers and ReverTra Ace (Toyobo), according to the manufacturer’s instructions. The NS5 coding regions were amplified using a pan-flavivirus FW/RV universal primer pair and DNA polymerase (KOD plus Neo, Toyobo), together with the resultant cDNA template from cells infected with the JEV porcine vaccine strain (m strain), distilled water (negative control), or the genome of Dengue virus 2 PDK53 (accession KU725664) (positive control), as described above, but with 40 amplification cycles. As an additional control for RNA extraction, the actin gene was amplified using the specific primer pair Act-2F (5′-ATGGTCGGYATGGGNCAGAAGGACTC-3′) and Act-8R (5′-GATTCCATACCCAGGAAGGADGG-3′) [62].

### Detection of the viral genome from mosquito homogenate

Next, the primer pair was used to detect the flavivirus genome in crude supernatants prepared from pooled mosquito homogenates containing the JEV vaccine strain (m strain). Mosquitoes (*Culex pipiens pallens*) were obtained from Sumika Technoservice Corporation (Hyogo, Japan). Mosquito pools (one pool comprised ten mosquitoes) were placed into 50 ml safe-lock microfuge tubes together with zirconia beads and crushed under freezing conditions. Next, 1 ml of PBS (without calcium/magnesium; PBS(-)) was added to the tubes containing the crushed mosquitoes, which were then homogenized on ice. The above-mentioned JEV vaccine strain, which was titrated in C6/36 cells to obtain the TCID_50_ (as described above), was diluted tenfold and mixed with each homogenized mosquito pool (comprising 10 mosquitoes), followed by centrifugation at 9000 × g (4 °C for 10 min). The final concentration of JEV in the crude supernatant was 10^5.5^–10^1.5^ TCID_50_/ml. A QIAamp Viral RNA Mini Kit (Qiagen, Hilden, Germany) was used to extract total RNA from the centrifuged homogenate containing JEV (140 µl of whole homogenate was used for RNA extraction; the final volume of extracted RNA was 60 µl). Single-stranded cDNA was synthesized from 10 µl of the extracted RNA and used for cDNA synthesis using random primers and Rever Tra Ace (Toyobo), according to manufacturer’s instructions. The NS5 coding regions were amplified (40 amplification cycles) using the pan-flavivirus FW/RV universal primer pair and DNA polymerase (KOD plus Neo), together with the cDNA template derived from the mosquito cell homogenate.

### Measurement of viral copy number by quantitative real-time PCR

In addition, we converted the virus titer in the spiked pooled mosquito samples into genomic copy number by performing quantitative real-time PCR (q-PCR) using cDNA synthesized from RNA extracted from a JEV vaccine strain (serially diluted tenfold from 10^5.5^–10^0.5^ TCID_50_/ml) and a standard plasmid encoding the JEV genome. Q-PCR was conducted using mix reagent (THUNDERBIRD®Probe qPCR Mix, Toyobo, Osaka) and the above-mentioned cDNA template derived from RNA extracted from the diluted JEV virus, or standard plasmids pEX-K4J2 (Eurofins Genomics) encoding the JEV NS5 region (Nakayama MY 2009 P578662), together with a TaqMan probe and primers (mFU1 and cFD2) targeting the NS5 gene [53]. The TaqMan probe comprised an oligonucleotide with a fluorescent reporter dye attached to the 5’ end, and a non-fluorescent quencher (NFQ) attached to the 3’ end. The sequence of the mFU1 (forward) primer is 5′-TACAACATGATGGGAAAGCGAGAGAAAAA-3′, and that of the cFD2 (reverse) primer is 5′-GTGTCCCAGCCGGCGGTGTCATCAGC-3′. The oligonucleotide sequence of the TaqMan probe is FAM-TCCGTGACATAGCAGGAAAGCAAG-NFQ. Next, quantitative real-time PCR was performed using the CFX96 Real-Time PCR System (Bio-Rad, Hercules, CA) and the following cycling conditions: initial denaturation at 95 °C for 1 min, followed by 40 amplification cycles (denaturation at 95 °C for 15 s and annealing/extension at 58 °C for 45 s). The copy number of the viral genome in each cDNA sample was calculated by reading off the Ct values for cDNAs from a standard curve constructed by plotting the copy number of a plasmid (serially diluted tenfold) encoding the JEV genome versus Ct value (Additional file 1: Fig. S1). The copy number of the viral genome was determined in quadruplicate. The concentration of plasmid encoding the JEV NS5 region (Nakayama MY 2009 P578662) was determined in a NanoDrop ND2000 Spectrophotometer (NanoDrop Technologies, Inc., Wilmington, DE) and converted to molecular copies using the following formula:

$${\text{Y copies}}/{\mu\text{l}} = {\text{X }}({\text{g}}/{\mu\text{l}}){\text{ DNA }} \times {\text{ 6}}.0{\text{2 }} \times {\text{ 1}}0^{{{\text{23}}}} /\left[ {{\text{DNA length }}\left( {{\text{bp}}} \right){\text{ }} \times {\text{ 2 }} \times {\text{ 33}}0} \right]$$

## Results

### Sequence alignment of NS5 regions and primer design

To design primer pairs that detect a broad range of flaviviruses, we performed multiple alignments of NS5 gene sequences from mosquito-borne, tick-borne, ISFs, and NKV flaviviruses. The multiple alignment identified an area of high homology between nucleotide sequences from individual strains of flaviviruses (Fig. 2), which enabled the design of a universal primer pair that detects flaviviruses broadly (pan-flavivirus FW/RV) (Table 1). To overcome some inevitable nucleotide variation (even in this highly homologous region among viral sequences), we gave mix bases such as “R”, “Y”, “H”, “B” or “N” within this primer pair. The nucleotide region bound by the pan-flavivirus RV primer overlaps that recognized by primers EMF1 and CFDJ9977 [54, 55, 58]; however, the lengths and nucleotide sequences of these primers are different from those of our novel primer.

### Ability of the universal primers to detect individual flavivirus genomes

**Fig. 3:**
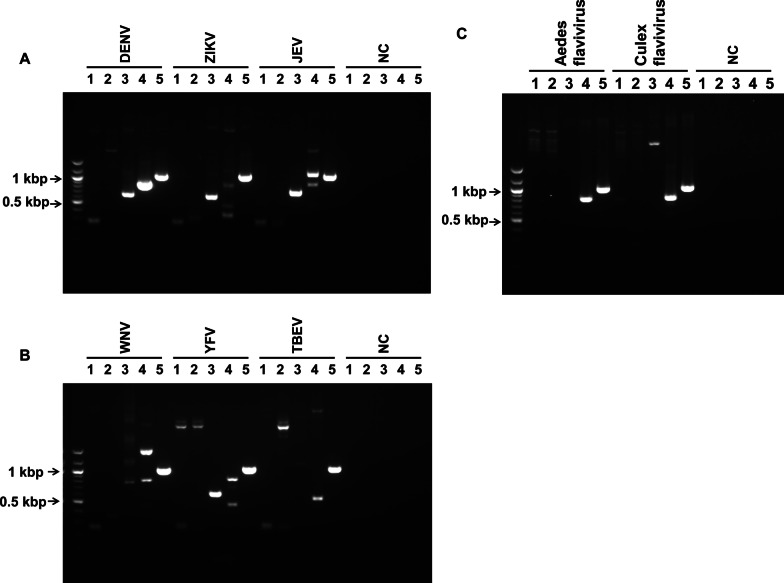
PCR assay using the universal primer pair to amplify the NS5 region. Artificially synthesized oligonucleotides encoding flavivirus NS5 sequences (see “Materials and methods”) were used as templates for the PCR assay. The viral strains detected in this assay are as follows: DENV: Dengue virus 2 PDK53 (accession KU725664); ZIKV: Zika virus mosquito/Haiti/1682/2016 (accession MF384325); JEV: Japanese encephalitis virus Nakayama/MY/2009/P578662 (accession HE861351) (A); WNV: West Nile virus BSL26-11 (accession JQ700442); YFV: YFV ES-504 (accession KY885000); TBEV: Tick-borne encephalitis virus C11-13 (accession KP644245) (B); Aedes flavivirus: Aedes flavivirus Narita-21 (accession AB488408); and Culex flavivirus: Culex flavivirus Tokyo (accession AB262759) (C). Lane 1, FU1/cFD2; lane 2, MA/cFD2; lane 3, EMF1/VD8; lane 4, FUDJ9166/CFDJ9977; lane 5, Pan-flavivirus FW/RV (this article). NC: negative control (distilled water). The bands represent amplicons of the NS5 region. The images shown are cropped from the whole gel images

To confirm the utility of the primer pair that we designed (pan-flavivirus FW/RV), we used it to amplify the NS5 region of eight different flaviviruses species. We conducted PCR to amplify artificially synthesized oligonucleotides (Type II DENV, ZIKV, JEV, WNV, YFV, TBEV, Aedes flavivirus, and Culex flavivirus) using pan-flavivirus FW/RV and four other primer pairs (FU1/cFD2, MA/cFD2, EMF1/VD8, and FUDJ9166/CFDJ9977) [54–58], which are used commonly to amplify flavivirus genome. As shown in Fig. 3, the primer pairs that were used generated amplicons of the following sizes: FU1/cFD2 and MA/cFD2, < 300 bp; EMF1/VD8, < 700 bp; and FUDJ9166/CFDJ9977 < 900 bp (except for the amplicon of JEV), all of which corresponded to the sizes reported in the literature [54–57]. The amplicon obtained by PCR using the pan-flavivirus FW/RV primer pair was approximately 1 kbp in size, which corresponds to the expected sizes described in “Primer design” in the “Materials and Methods”. However, except for pan-flavivirus FW/RV, the other primer pairs did not necessarily amplify the NS5 region of all flavivirus species tested. Indeed, FU1/cFD2 amplified Type II DENV, ZIKV, JEV, WNV, YFV, and TBEV, but not ISFs. MA/cFD2 amplified ZIKV, JEV, YFV (weak band) and TBEV (although the product of ZIKV was not of the expected size), but no other species of flavivirus tested. EMF1/VD8 amplified Type II DENV, ZIKV, JEV, and YFV, but no other species tested. FUDJ9166/CFDJ9977 amplified Type II DENV, JEV, WNV, YFV, and ISFs (although the product of JEV was not of the expected size), but not ZIKV or TBEV. In addition, FUDJ9166/CFDJ9977 amplified some additional bands from the genomes of WNV and YFV. The unexpected band observed in the JEV sample is likely an extra-band; this band is larger than the expected size (841 bp) obtained from the template (JEV Nakayama MY 2009 P578662), suggesting that it could be the result of poor annealing to the target sequence. By contrast, pan-flavivirus FW/RV amplified all of the viral genomes and did not generate any non-specific bands. The intensity of the band for each amplicon produced by each primer pair was also different (Fig. 3). The intensity of the PCR products generated by FUDJ9166/CFDJ9977 and pan-flavivirus FW/RV was high; however, the intensity of those generated by the other primer pairs (i.e., FU1/cFD2, MA/cFD2, and EMF1/VD8) were not as high as that generated by pan-flavivirus FW/RV, indicating that these primer pairs are not the preferred choice for further analyses such as gene sequencing. To confirm whether the amplicons generated by the novel universal FW/RV primer are appropriate for gene sequencing, we sequenced the PCR products. The results showed that individual nucleotide sequences, excluding the PCR primers and unread regions at both ends of the PCR products, were 100% identical to the sequences of the templates (Type II DENV (accession KU725664), ZIKV (accession MF384325), JEV (accession HE861351), WNV (accession JQ700442), YFV (accession KY885000), TBEV (accession KP644245), Aedes flavivirus (accession AB488408), and Culex flavivirus (accession AB262759)).

### Amplification of the NS5 region of JEV from infected cells

**Fig. 4:**
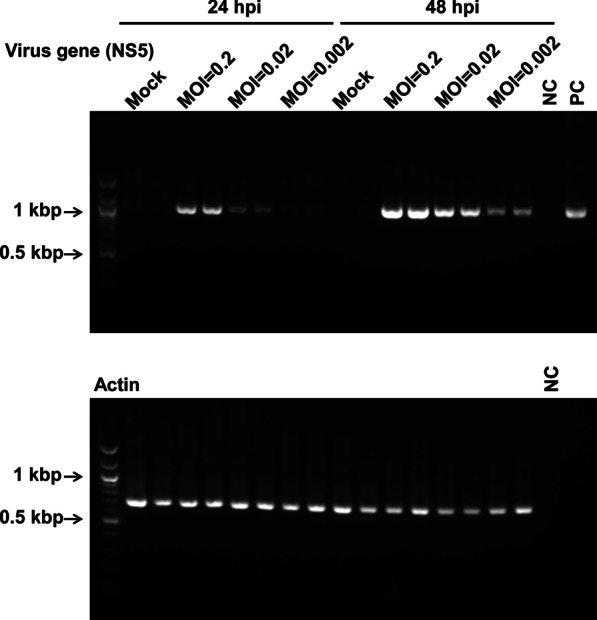
Amplification of the NS5 coding region of JEV from infected cells. NS5 coding sequences were amplified by PCR of cDNA templates derived from infected cells. Mosquito cell line (C6/36) was infected with JEV porcine vaccine strain at a multiplicity of infection of 0.2, 0.02, or 0.002. After 24 or 48 h, infected cells were collected and prepared for RT-PCR (see *“Materials and Methods”*). Mock: mock-infected cells (cells inoculated with PBS); NC: negative control (distilled water); PC: positive control (the genome of Dengue virus 2 PDK53, accession KU725664 [as used in Fig. 3]). The bands represent amplicons of the NS5 region (upper panel) and actin (lower panel). The images shown are cropped from the whole gel images

Next, we amplified the viral NS5 region from cells infected with live flavivirus. The mosquito cell line C6/36 was infected with the JEV vaccine strain at an m.o.i. of 0.2, 0.02, and 0.002, or mock-infected with PBS (−). Infected cells were collected, cDNA was synthesized from extracted RNA, and RT-PCR was performed using the universal primer pairs together with synthesized cDNA, distilled water (negative control), or the genome of DENV 2 PDK53 (accession KU725664) (positive control). Specific bands corresponding to the expected amplicon size of JEV were detected in the PCR assay using cDNA templates synthesized from cells at 24 h post-infection, although the intensity of each band was different; the intensity of the bands derived from cells infected at an m.o.i. of 0.2 was high, that from cells infected at an m.o.i. of 0.02 was modest, and that from cells infected at an m.o.i. of 0.002 was low (Fig. 4). These band intensities were higher when the PCR assay was performed using cDNA templates from the cells at 48 h post-infection, although again the intensities differed according to the m.o.i. No bands were observed under mock infection conditions (Fig. 4), suggesting that the primers were specific.

### Amplification of the NS5 region of JEV from crude mosquito fluid

**Fig. 5:**
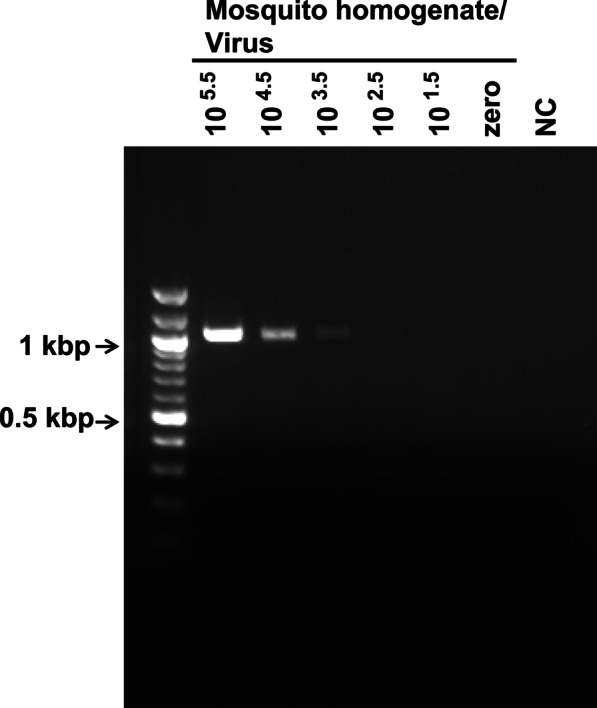
Amplification of the NS5 coding region of JEV from crude mosquito homogenates. NS5 coding sequences were amplified by PCR using cDNA templates derived from crude mosquito homogenates. The mosquito homogenate was prepared from pooled mosquito samples (10 mosquitoes per sample) and then spiked with the serially diluted JEV vaccine strain. The virus titer in the mosquito homogenate was 10^5.5^–10^1.5^ TCID_50_/ml or zero (no virus). NC: negative control (distilled water). cDNA was synthesized from RNA extracted from an aliquot of whole mosquito fluid. The bands represent amplicons of the NS5 region. The image shown is cropped from the whole gel image

Finally, to confirm the utility of the novel primer pairs for amplification of the viral NS5 region from crude insect samples, we tested them using crude homogenates derived from pooled mosquito samples (10 mosquitoes per sample) spiked with different concentrations of the JEV vaccine strain (the virus titer in mosquito homogenates was 10^5.5^–10^1.5^ TCID_50_/ml). Briefly, cDNA was synthesized from RNA extracted from mosquito homogenates (including JEV), and RT-PCR was conducted using the primer pair; distilled water was used as a negative control (the RT-PCR conditions were the same as those used to amplify virus sequences from the infected mosquito cell line C6/36). Specific bands corresponding to the virus genome were detected in mosquito homogenate samples, with different samples showing different band intensities (Fig. 5). In the case of RNA extracted from the pooled mosquito sample, the detection limit was 10^2.5^ TCID_50_/ml. We also converted the virus titer into viral genome copy number by performing qPCR using cDNA synthesized from RNA extracted from the JEV vaccine strain (serially diluted tenfold from 10^5.5^–10^0.5^ TCID_50_/ml) together with a standard plasmid encoding the JEV genome (Additional file 1: Fig. S1). The qPCR assay revealed that the detection limit in crude samples was 10^2.5^ TCID_50_/ml (equivalent to 10^4.92^ copies/ml of mosquito homogenate), which corresponds to 10^–0.43^ TCID_50_ (equivalent to 10^1.99^ copies) for a single reaction in the PCR (Table 2).

**Table 2:** Amount of infectious virus particles or virus genome copies spiked into the pooled mosquito sample and the total viral load used in the PCR reaction

Infectious virus particles/virus genome copies					
Viral titer in the mosquito homogenate	10^5.5^	10^4.5^	10^3.5^	10^2.5^	10^1.5^
(TCID_50_/ml)^a^					
Viral genome in the mosquito homogenate	10^7.89^	10^6.72^	10^5.95^	10^4.92^	10^4.20 c^
(copies/ml)^b^					
Infectious virus load used in the PCR reaction	10^2.57^	10^1.57^	10^0.57^	10^–0.43^	10^–1.43^
(TCID_50_/PCR reaction)^d^					
Viral genome used in the PCR reaction	10^4.95^	10^3.78^	10^3.02^	10^1.99^	10^1.27^
(copies/PCR reaction)^d^					

^a^The JEV vaccine virus was spiked into the 10 mosquito homogenate to yield final concentrations ranging from 10^5.5^–10^1.5^ (tenfold dilutions)

The titer of the spiked virus was determined based on TCID_50_ (see “Materials and Methods”)

^b^The number of copies of the viral genome spiked into the mosquito homogenate was based on the viral genome copy number of cDNAs synthesized from RNA extracted from the JEV vaccine strain (serially diluted from 10^5.5^–10^0.5^ TCID_50_/ml). The values represent the mean of quadruplicate measurements. The copy number of the viral genome in each cDNA sample was calculated by reading off the cDNA Ct values from a standard curve constructed by plotting the copy number of a plasmid (serially diluted) encoding the JEV genome versus Ct value (see Additional file 1: Fig. S1)

^c^This value represents the mean of triplicate measurements (due to the detection limit)

^d^The viral load used in the PCR reaction was calculated according to the amount of RNA extracted from the mosquito homogenate and used for cDNA synthesis

## Discussion

Here, we report three main findings with respect to detection of flaviviruses using a novel universal primer pair. We show that: (i) the primer pair amplified the NS5 coding region of all flavivirus species tested; (ii) the primer pair detected the viral genome specifically in cells infected with live flavivirus; and (iii) the primer pair detected the viral genome in crude mosquito homogenates.

Of the primer pairs used in this study, the pair that we designed (pan-flavivirus FW/RV) was the only one able to amplify the NS5 region of all eight species of flavivirus tested; these viruses encompass all major subgroups of mosquito-borne flaviviruses, tick-borne flaviviruses, and ISFs. By contrast, amplification by other primer pairs (FU1/cFD2, MA/cFD2, EMF1/VD8, and FUDJ9166/CFDJ9977) was limited; in particular, they did not amplify the NS5 region of some recently identified flavivirus strains, including ISFs, even though the nucleotide sequences of EMF1 and CFDJ9977 overlap with a region that is also targeted by our pan-flavivirus RV. These results may be because the other primer pairs were designed in the 1990s; therefore, they are unlikely to detect recent strains (the flaviviruses used as oligonucleotide templates for the PCR assays conducted herein were isolated mainly in the 2000s). In addition, classical ISFs identified mainly in the 2000s [7–23] (the exception is the ISF prototype “cell fusion agent virus”, which was discovered in 1975) [63] show large genetic differences from the major subgroups of mosquito-borne or tick-borne flaviviruses. These genetic differences mean that primer pairs FU1/cFD2, MA/cFD2, EMF1/VD8, and FUDJ9166/CFDJ9977, which were designed in the 1990s, are unlikely to detect classical ISFs because they do not recognize gene sequences recently identified as “insect-specific flaviviruses” [7–32].

The PCR assay using the pan-flavivirus FW/RV primers yielded bands that were larger in size and higher in intensity than the other well-known primer pairs used in the same assay (Fig. 3 and the Table 1). The fact that the primer pair we designed produces a fragment large enough for nucleotide sequencing means that this primer pair is a potentially powerful tool for genetic analysis of both known and novel flavivirus strains. A recent review by Blitvich and Firth [2] suggests that some flaviviruses may be novel ISF strains; however, identification of some novel strains was based on ambiguous analyses using inadequate information obtained from genome sequences of limited size (< 300 bp), which are too short for reliable comparison. It is proposed that flaviviruses possessing > 84% nucleotide sequence identity should be classified within the same species [56]. Therefore, in some cases short nucleotide sequences could misidentify “novel species” based on low (< 84%) nucleotide sequence similarity. Thus, to avoid erroneous classification, nucleotide sequences of appropriate length are needed for reliable analyses. The novel universal primer pair will allow reliable genetic analyses because it generates a product > 1000 bp in length, which is suitable for sequence analyses; this property increases the utility of this novel primer pair.

Global detection of flaviviruses in field samples requires a primer pair specific enough to detect the target sequences in the cell lysate. The PCR assay using cDNA templates synthesized from total RNA samples derived from infected mosquito cells revealed that the novel universal primer pair generated specific bands corresponding to the NS5 gene sequences of JEV. In addition, the intensity of the bands produced by pan-flavivirus FW/RV was dependent on the infection conditions (i.e., the m.o.i., the incubation time, and analysis time post-infection), suggesting that the intensity of individual bands reflects the amount of template (copy number) available. This result led us to surmise that our PCR system and specific primer pair can detect not only artificially synthesized oligonucleotides but also cDNA templates prepared from mosquito or tick cell lysates, which harbor variable flavivirus copy numbers. To prove this hypothesis, we used the new primer pair to detect the viral genome in crude mosquito homogenates. The detection limit in samples derived from mosquito homogenate was 10^2.5^ TCID_50_/ml (equivalent to 10^4.92^ copies/ml of mosquito homogenate or 10^1.99^ copies/PCR reaction); these parameters are consistent with a recent report showing that the viral genome is preserved in wild mosquitoes. Martin et al. [64] examined the viral load of the cell fusion agent virus, which is a species of ISF, in individual mosquitoes trapped in the field. The viral load in males ranged from 1.25 × 10^2^–5.50 × 10^6^ RNA copies (median > 10^6^ copies) per mosquito, whereas that in females ranged from 5.42 × 10^3^–8.70 × 10^6^ RNA copies (median, > 10^6^ copies) per mosquito (although the viral load in gravid females was lower [3.29 × 10^2^–3.53 × 10^6^ RNA copies per mosquito; median 10^4^ copies]). Based on the parameters reported by Martin et al., and the detection limit of JEV in our experiment, the novel universal primer pair is likely to detect virus in field-collected samples, although the detection limit may differ depending on the type of flavivirus present in the sample. One limitation of this study is that only JEV was used to determine the detection limit of the assay for the flavivirus genome. Further studies are required to determine the detection limit for viruses other than JEV, and to actually detect flaviviruses in field-collected samples.

The present results showing the specificity and detection threshold of the novel universal primer pair suggest that our novel primer pair could efficiently detect flavivirus at levels comparable with those identified in wild mosquitoes, making it a powerful tool for detecting flavivirus genomes in whole arthropod (mosquito and likely tick as well) homogenates.

## Conclusion

Globalization and global warming mean that flavivirus-mediated diseases are a public health concern in many parts of the world. In addition, we are unsure about the potential of novel flaviviruses such as ISFs to evolve into major human pathogens. Also, some ISFs can affect the amounts of human-pathogenic virus harbored by arthropods; e.g., superinfection experiments suggest that some ISFs suppress or support replication of mosquito-borne flaviviruses [19, 33–36]; if this is the case, then ISFs may have an indirect effect on public health. Therefore, it is important to investigate the prevalence of flaviviruses, including novel strains or novel virus groups such as ISFs, in arthropods. This will allow risk assessments regarding possible flavivirus-mediated diseases. It will also increase our understanding of which species have positive or negative effects on replication of other viruses. In addition, unknown flavivirus species are likely to be discovered in hematophagous arthropods in the future. Some of these may even be the cause of diseases of unknown origin. Here, we developed genus-specific universal primers that may make it possible to amplify NS5 sequences from a broad spectrum of flaviviruses belonging to different genetic subgroups. This primer pair will allow us to better investigate the prevalence of flaviviruses in arthropods worldwide, improve public health and safety, and increase our understanding of flavivirus ecology.

## Supplementary Information

**Additional file 1. Fig. S1**: Standard curve for the quantitative-PCR assay. A standard curve of Ct values versus copy number. The standard curve was constructed by 10-fold serial dilution (8.16 × 10^7^ to 8.16 copies) of a plasmid encoding the JEV genome. All Ct values are the mean values of quadruplicate measurements, except for the value of 8.16 copies, which is the mean value of triplicate measurements (due to the detection limit).

## Data Availability

All data generated or analyzed during this study are included in the published article and its supplementary figure.
