# Supplementary figures and images for: Development of genus-specific universal primers for the detection of flaviviruses
Source: Virol J. 2021 Sep 15;18:187. doi: 10.1186/s12985-021-01646-5 (PMC8442469; doi:10.1186/s12985-021-01646-5)

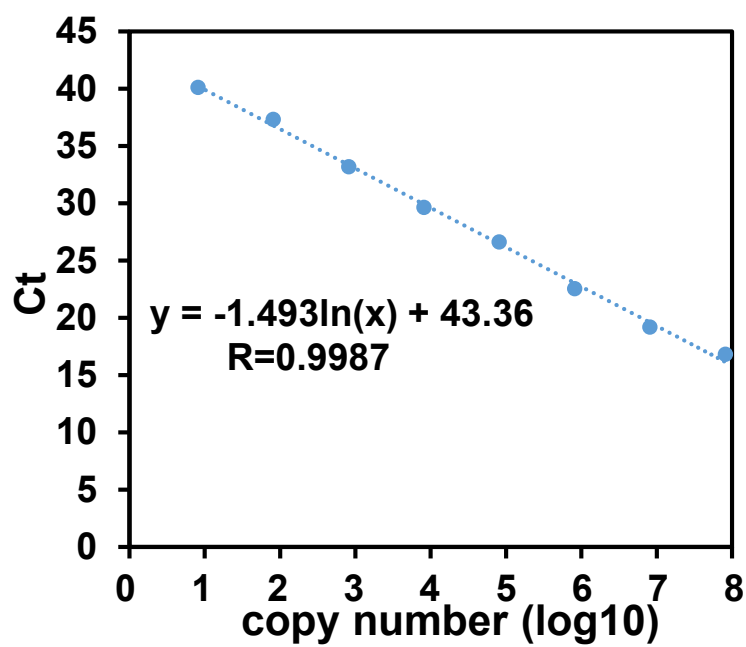

Figure S1.

Supplement: Supplementary file 1 — Additional file 1. Fig. S1: Standard curve for the quantitative-PCR assay. A standard curve of Ct values versus copy number. The standard curve was constructed by 10-fold serial dilution (8.16 × 10^7^ to 8.16 copies) of a plasmid encoding the JEV genome. All Ct values are the mean values of quadruplicate measurements, except for the value of 8.16 copies, which is the mean value of triplicate measurements (due to the detection limit). [file 12985_2021_1646_MOESM1_ESM.pdf]
